# Effect assessment of evidence-based nursing in combination with clinical nursing pathway on nephrotic syndrome care in children: A protocol for systematic review and meta-analysis

**DOI:** 10.1097/MD.0000000000025990

**Published:** 2021-06-04

**Authors:** Xia Yu, Cai-Yan Han

**Affiliations:** a Department of Pediatrics; b Department of Neurology, Hanchuan People's Hospital, Hanchuan, Hubei Province, China.

**Keywords:** children, clinical nursing pathway, evidence-based nursing, nephrotic syndrome care

## Abstract

**Background:**

Childhood nephrotic syndrome is widespread in pediatric nephrology. In most cases, it needs hospitalization for patient management. An increasing number of studies report that proper nursing care could promote the rate of treatment and improve post-treatment prognosis. Clinical nursing pathways refer to innovative nursing modes with high-quality, excellent efficacy, and low costing treatment. There are reports on how nursing methods that utilize data combine with clinical nursing pathway to enhance nephrotic syndrome care in kids. However, the results remain controversial. Therefore, it is necessary to conduct this study to systematically explore how evidence-based nursing combined with clinical nursing pathway plays a role in nephrotic syndrome care among children.

**Methods:**

This study protocol will conduct a comprehensive search on MEDLINE, Cochrane Library, CINAHL, EMBASE, Scopus, Chinese National Knowledge Infrastructure, WanFang, and Web of Science electronic databases to identify relevant research articles from inception to April 25, 2021. Studies in both English and Chinese languages are used for this study. This study protocol will analyze randomized controlled trials that investigated the role of evidence-based nursing combined with clinical nursing pathway to care for nephrotic syndrome in children. Two authors will independently screen the search results, select suitable studies for inclusion, extract the characteristics and outcome data of the selected studies, and evaluate the risk of bias based on standard Cochrane methodology. Any discrepancies will be resolved by consensus.

**Results:**

The present study will summarize high-quality evidence to systematically explore how a nursing model based on evidence combined with clinical nursing pathway influences the caring of children with nephrotic syndrome.

**Conclusion:**

The present study will summarize the direct and indirect evidence to judge whether evidence-based nursing combined with clinical nursing pathway can improve the treatment and post-treatment prognosis in children with nephrotic syndrome.

**Ethics and dissemination:**

This study does not require an ethical approval.

**Registration number:**

April 25, 2021.osf.io/bcrdk/ (https://osf.io/bcrdk/).

## 1 Introduction

Nephrotic syndrome is a commonly occurring kidney disease in children. It is prevalent all over the world, with an incident rate of 1.5 of 16.9 patients for every 100,000 kids and a prevalence of 16 patients for every 100,000 children.^[1–3]^ It is the most prevalent congenital malformations of the kidney and the urinary tract. Nephrotic syndrome is also the second most encountered kidney-related condition in pediatric nephrology clinic. In general, based on the preliminary response to corticosteroid therapy at inspection, children with nephrotic syndrome are typically categorized as steroid-sensitive nephrotic syndrome and steroid-resistant nephrotic syndrome. The categorization is based on the International Study of Kidney Disease in Children studies during the 1970s, which indicated that regardless of morphological variations on kidney biopsy, the response to corticosteroid and additional immunosuppressive treatments is the most critical prognostic indicator in nephrotic syndrome.^[4]^ Therefore, as long as therapy remain responsive, kids with steroid-sensitive nephrotic syndrome exhibit outstanding prognosis with more than 95% improbability to advance to the final phase of kidney disease.^[5]^

Clinical nursing pathway is widely considered as a means that provide useful knowledge regarding distinct patients and their treatment, including the provision of straightforward assistance in clinical settings. Therefore, it is an innovative high-quality nursing mode with excellent efficacy and affordability.^[6,7]^ A previous study showed that adopting a mental health clinical nursing pathway to help patients with malignant tumors can effectively reduce the suicidal intentions in patients and enhance their life standard.^[8]^ As the body of knowledge associated with nursing based on evidence grows and as nursing practice becomes increasingly reliant on actual, reliable scientific evidence from the patients, the conventional limited empiricism nursing model is evolving into a novel nursing concept.^[9]^ Few studies have reported the impact of utilizing nursing models based on evidence combined with clinical nursing pathway to care for children with nephrotic syndrome. However, the results are controversial. Thus, the current study will systematically explore the role of evidence-dependent nursing combined with clinical nursing pathway to care for nephrotic syndrome children.

## 2 Objectives

The present study aims to explore the role of evidence-based nursing combined with clinical nursing pathway to care for children with nephrotic syndrome.

## 3 Methods

### 3.1 Study registration

The present study will be performed as per the Preferred Reporting Items for Systematic Review and Meta-Analysis Protocols,^[10]^ and it has been listed on the Open Science Framework (OSF, http://osf.io/).

### 3.2 Eligibility criteria for included studies

Ritu

### 3.3 Types of participants

Children (age, <18 years) with a confirmed diagnosis of nephrotic syndrome will be included; there will be no restrictions on gender, race, and country.

#### 3.3.1 Types of participants

Patients in the experimental group must receive evidence-based nursing intervention combined with clinical nursing pathway. Meanwhile, patients in the comparisons group must only receive evidence-based nursing intervention, clinical nursing pathway only, or no nursing intervention, except evidence-based nursing intervention combined with clinical nursing pathway.

#### 3.3.2 Types of outcomes

The major outcomes included duration of hospitalization, hospitalization fees, and the satisfaction level of patients.

#### 3.3.3 Types of studies

This protocol study will include randomized control trials investigating the role of combining evidence-based nursing and clinical nursing pathway to treat nephrotic syndrome children.

### 3.4 Search methods for identification of studies

#### 3.4.1 Electronic searches

A comprehensive search will be performed on MEDLINE, Cochrane Library, CINAHL, EMBASE, Scopus, Chinese National Knowledge Infrastructure, WanFang, and Web of Science electronic databases to identify relevant studies from their inception to April 25, 2021. Articles published in both English and Chinese will be selected. The search terms given below will be combined using Boolean logic (AND, OR, or NOT) to identify relevant studies: “clinical pathway,” “evidence-based nursing,” “nephrotic syndrome,” “randomized controlled trial,” and “RCT.”

#### 3.4.2 Search other sources

This study protocol will go through the reference lists of all selected primary studies and review articles to obtain other related references.

### 3.5 Data collection and analysis

#### 3.5.1 Study selection

**Figure 1:**
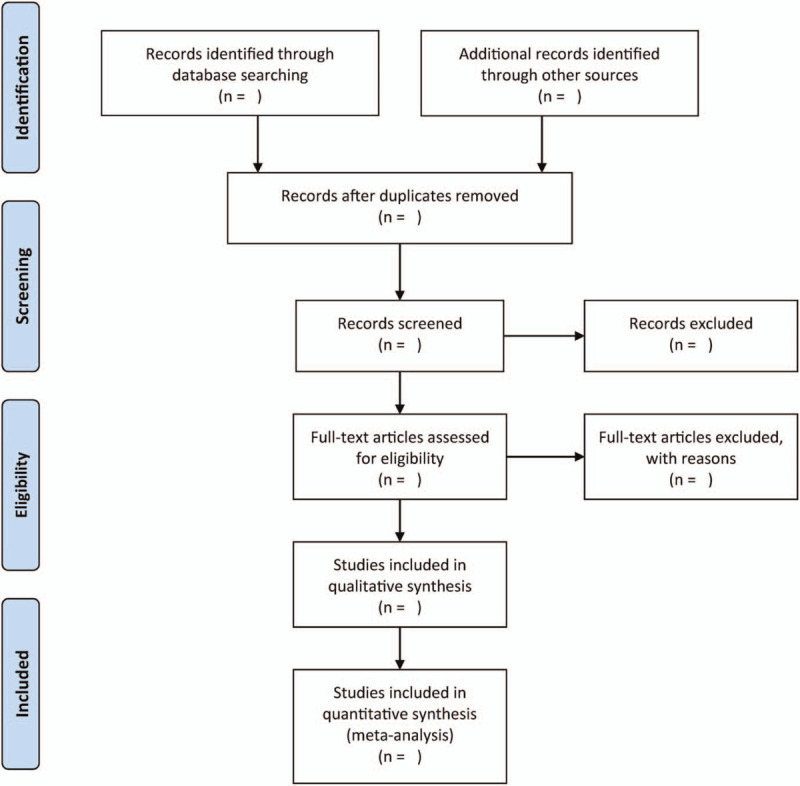
Flowchart of study selection.

A couple of authors will autonomously study the titles/abstract obtained through electronic and manual exploration. Afterwards, the complete text of each study will be further scrutinized to identify relevant studies. The present study will resolve discrepancies in judgement by consensus and discussion. The flowchart of searching and screening studies will be presented in Figure 1.

#### 3.5.2 Data extraction and management

A pair of independent authors will extract all relevant information from each study through a generalized data extraction form. The following items will be extracted from individual studies: appropriate characteristics of the studies (e.g., first author name, publication year, and country), intervention characteristics (e.g., the name of nursing intervention, intervention time, duration time, and follow-up time), population characteristics (e.g., nephrotic syndrome disease diagnosis, age, gender, ethnicity, and number of participants), and outcome measures. All discrepancies in judgement will be resolved through consensus and discussion.

#### 3.5.3 Assessment of risk of bias in included studies

A pair of independent authors will evaluate the bias risk in the included studies. The procedure will be based on the Cochrane Risk of Bias Tool.^[11]^ This study will resolve discrepancies in judgement by consensus and discussion.

#### 3.5.4 Measures of treatment effect

This protocol study will analyze the continuous data according to mean differences or standardized mean differences and 95% confidence interval, while the dichotomous data will be analyzed using the relative risk and 95% confidence interval.

#### 3.5.5 Dealing with missing data

In cases of missing data, the corresponding authors will be contacted to collect the lost data.

#### 3.5.6 Assessment of heterogeneity

This study protocol will utilize the *I*^2^ statistic to evaluate statistical heterogeneity across studies. It is intended to consider a heterogeneity level of above 50% as substantial or high, in which case the random-effects model will be utilized; otherwise, the fixed-effects model will be adopted.

#### 3.5.7 Sensitivity analysis

We will intend to repeat the analyses while excluding studies at high risk of bias to evaluate the robustness of our findings if applicable.

#### 3.5.8 Assessment of reporting biases

It is also planned to devise and assess a funnel plot to investigate any plausible small study and publication bias where applicable. It is also intended to evaluate for asymmetry through Egger's test.

## 4 Discussion

The clinical nursing pathway involves the devising and application phases. In a previous research, a specialized nursing team devised a nursing path which included the senior level to the visiting physician, head nurse, and nurses.^[12]^ The primary reference was local and foreign-associated studies and the explicit settings of the patients in the chosen group. The application of the nursing model must be performed strictly in accordance with the devised strategy. Afterwards, the completed section should be specified, while the incomplete section institutes the primary content of the subsequent nursing phase.^[13]^ Clinical nursing pathway aims to enhance the quality of treatment for patients by enabling multidisciplinary partnership and leading health care practitioners with evidence-based treatment strategies. The present study will summarize high-quality evidence to systematically explore how nursing based on evidence combined with clinical nursing pathway to care for children with nephrotic syndrome, besides facilitating the development of clinical guidelines, nurses can use the work done here as a reference. To the best of the author's knowledge, this study is the first to evaluate the role of evidence-based nursing combined with clinical nursing pathway when used to provide care for children with nephrotic syndrome.

## Author contributions

**Conceptualization:** Xia Yu.

**Data curation:** Xia Yu, Cai-Yan Han.

**Investigation:** Cai-Yan Han.

**Formal analysis:** Xia Yu, Cai-Yan Han.

**Funding acquisition:** Cai-Yan Han.

**Methodology:** Xia Yu, Cai-Yan Han.

**Project administration:** Cai-Yan Han.

**Resources:** Cai-Yan Han.

**Software:** Xia Yu.

**Validation:** Xia Yu, Cai-Yan Han.

**Visualization:** Xia Yu, Cai-Yan Han.

**Writing – original draft:** Xia Yu, Cai-Yan Han.

**Writing – review & editing:** Cai-Yan Han.
